# Report of the "Lancet" Special Commission on the Relative Strengths of Diphtheria Antitoxic Serums

**Published:** 1897-06

**Authors:** 


**Report of the "Lancet" Special Commission on the Relative Strengths of Diphtheria Antitoxic Serums.** Pp. 59. London: The Offices of the *Lancet*. 1896.

This report, reprinted from the *Lancet*, deals with a subject of considerable importance to those medical practitioners wishing to make use of antitoxic serum in the treatment of diphtheria. Some at least of the disfavour shown in some quarters towards the adoption of this treatment in England may be ascribed to the difficulty in determining the quality and strength of the various makes of serum, a point of considerable urgency seeing that its efficiency depends in great part upon the strength of dose in relation to the early or late period of the disease. "We should appreciate at once," state the Commissioners, "the importance of the fact that a regiment of soldiers had been marched into a malarial district with a supply of quinine which had been so adulterated that the medical man was really introducing only one-fourth the quantity of the drug which he had prescribed owing to the presence of inert material. In the present state of our knowledge it is not too much to say that the influence exerted by the exhibition of antitoxin on diphtheria is at any rate quite as marked as that exerted by quinine on malaria." In any case, it is obviously of great importance to have some trustworthy information as to the strength of our drugs, and such information it is the aim of this report to supply.

